# Stem Cells from Human Exfoliated Deciduous Teeth Attenuate Trigeminal Neuralgia in Rats

**DOI:** 10.1155/2021/8819884

**Published:** 2021-01-18

**Authors:** Xiaofeng Bai, Xuedi Zhang, Chun Wang, Yao Liu, Xuemei Liu, Yu Fan, Xia Zhang

**Affiliations:** ^1^Department of Oral and Maxillofacial Surgery, Hospital of Stomatology, China Medical University, 117, N, Nanjing Street, Shenyang, China; ^2^Liaoning Provincial Key Laboratory of Oral Diseases, 117, N, Nanjing Street, Shenyang, China; ^3^Department of Anesthesiology, Hospital of Stomatology, China Medical University, 117, N, Nanjing Street, Shenyang, China; ^4^Department of Pediatric Dentistry, Hospital of Stomatology, China Medical University, 117, N, Nanjing Street, Shenyang, China; ^5^Department of Pathology, Hospital of Stomatology, China Medical University, 117, N, Nanjing Street, Shenyang, China

## Abstract

Trigeminal neuralgia is an incurable progressive nervous system disease that can last for several months or years. Stem cells from human exfoliated deciduous teeth (SHED) are a candidate source for cell-based therapy. Owing to their neuroprotective and immunomodulatory effects, these neural crest cells have potential roles in mediating chronic pain. In this study, we established a rat model of chronic constriction injury of the infraorbital nerve (CCI-ION) to evaluate the analgesic effect of SHED in neuropathic pain. The effects of local SHED transplantation on inflammatory cell infiltration in the trigeminal nerve were investigated based on hematoxylin and eosin staining. The levels of proinflammatory factors in the injured nerve and transient receptor potential vanilloid type 1 (TRPV1) expression in the trigeminal nerve and ganglion were quantified. The data showed that systemic or local injection of SHED attenuated the sensitivity of rats to mechanical stimuli after nerve injury, and this effect lasted throughout the observation period of 8 weeks. PKH26-labeled SHED were distributed to the ipsilateral trigeminal ganglions 24 and 72 hours after local injection. SHED transplantation at the lesion site led to reduced inflammatory cell infiltration and proinflammatory cytokine levels in the injured nerve and inhibited CCI-ION-induced upregulation of TRPV1 expression in the trigeminal nerve and ganglion in the early phase. Therefore, these results provide preclinical evidence that supports the use of SHED in the treatment of trigeminal neuralgia and potentially other chronic pain conditions.

## 1. Introduction

Trigeminal neuralgia is caused by lesions or injuries of the trigeminal nerve and can last for several months or years. Typical trigeminal neuralgia is characterized by unilateral, lancinating, spontaneous, or triggerable, often shock-like facial pain, with pain-free intervals. Trigeminal neuralgia is a progressive nervous system disease [1]. The treatment of trigeminal neuropathic pain is a clinical challenge, because the pathogenesis is very complex, involving structural and neurophysiological changes in the nervous system.

Stem cells from human exfoliated deciduous teeth (SHED) are collected from naturally exfoliated deciduous teeth by noninvasive procedures [2]. SHED possess the capacity to differentiate into various types of cells, including neural, osteogenic, and adipogenic cells [3]. For example, SHED have been shown to regenerate whole dental pulp with blood vessels and nerves in both patients and animals after implantation into injured teeth [4]. As neural crest-derived stem cells, SHED express various neural cell markers, such as glutamic acid decarboxylase, NeuN, nestin, glial fibrillary acidic protein, *β*III-tubulin, and neurofilament M [2, 5]. SHED can differentiate into dopaminergic neuron-like cells *in vitro*, and SHED transplantation partially improved apomorphine-induced rotational behavior in a rat model of Parkinson's disease [6]. In a rat model of spinal cord injury, SHED were shown to release key neurotrophic factors, suppress the apoptosis of astrocytes and neurons, and promote locomotor recovery [7]. These evidences support that SHED may be an attractive cell source for nerve regeneration treatments. Moreover, SHED possess superior immunomodulatory properties and reduce proinflammatory cytokine levels in immune disorders, such as systemic lupus erythematosus (SLE) [8]. The immune system interacts with the nervous system to regulate pain sensitivity. As neural crest cells, besides neuroprotective and immunomodulatory properties, SHED potentially play a role in mediating chronic pain. However, little information is available regarding the potential analgesic effects of SHED.

Transient receptor potential vanilloid type 1 (TRPV1) is a member of the transient receptor potential family of cation channels and can be activated by heat, acid, and ligands, such as capsaicin. In animals and humans, TRPV1 is localized in the peripheral and central nervous systems and has been demonstrated to play a key role in mediating neuropathic pain, visceral pain, inflammatory pain, and other forms of allodynia [9–11]. For example, TRPV1 expression in the trigeminal ganglia was elevated following tooth movement pain, and a TRPV1 antagonist reportedly alleviated orthodontic pain in rats [12].

In the present study, we established a rat model of trigeminal neuropathic pain by inducing chronic constriction injury of the infraorbital nerve (CCI-ION) to evaluate the effect of SHED on neuropathic pain. We aimed to provide evidence that SHED increase the mechanical threshold in trigeminal neuralgia rats. In addition, we investigated whether analgesic effects of SHED are involved in inflammatory cell infiltration and in regulating the levels of proinflammatory cytokines, such as tumor necrosis factor- (TNF-) *α* and interleukin- (IL-) 1*β*. Further, we confirmed that the long-term antihyperalgesic effect of SHED was associated with TRPV1 expression in the trigeminal nerve and trigeminal ganglion (TG).

## 2. Materials and Methods

### 2.1. SHED Isolation and Culture

Human exfoliated deciduous incisors were obtained as discarded biological samples from 6-to-8-year-old children in the Hospital of Stomatology, China Medical University, per the Institutional Review Board guidelines. Informed consent was obtained from the children's parents. The pulp was separated from the exfoliated teeth and digested in a solution of 4 mg/mL dispase (Boehringer Ingelheim, Mannheim, Germany) and 3 mg/mL collagenase type *Ι* (Worthington Biochemical Co., Lakewood, CO, USA) at 37°C for 30 min. Single-cell suspensions were obtained by passing the digest through a 70 *μ*m strainer (Merck Millipore Ltd., Cork, Ireland). The cells were cultured in alpha modification of Eagle's medium (HyClone, Logan, UT, USA), 15% fetal bovine serum (ExCell Bio, Shanghai, China), 100 mmol/L L-ascorbic acid (Sigma-Aldrich, St. Louis, MO, USA), and 100 U/mL penicillin-streptomycin (HyClone) at 37°C in the presence of 5% CO_2_. SHED of passages 3–5 were used in the experiments. Serum-free culture medium was used as the cell suspension medium for transplantation.

### 2.2. Surface Marker Expression Analysis

Cell surface markers were detected by flow cytometry (BD FACSAria II, Becton-Dickinson, Islandia, NY, USA). The primary antibodies used (anti-CD73, anti-CD105, anti-CD34, and anti-CD45) were purchased from BD Biosciences (Franklin Lakes, NJ, USA). Stem cells were adjusted to 1 × 10^6^/mL and incubated with the respective primary antibodies on ice for 30 min.

### 2.3. *In Vitro* Neurogenic and Osteogenic Differentiation Assays

Neurogenic and osteogenic differentiation was induced according to a previous report [2]. For neurogenic differentiation, cells were cultured in medium containing B27 supplement, 20 ng/mL basic fibroblast growth factor, 20 ng/mL epidermal growth factor, and 1% penicillin. After 4 weeks of culture, the SHED were fixed in 4% formaldehyde and analyzed for expression of the neural cell marker *β*III-tubulin by immunocytofluorescence staining (Sigma-Aldrich). Nuclei were stained with 4′,6-diamidino-2-phenylindole (DAPI, Dalian Meilun Biotechnology, Dalian, China). For osteogenic differentiation, SHED were cultured to 80% confluence and then incubated in medium supplemented with 10 nM dexamethasone and 1.8 mM monopotassium phosphate. After 4 weeks of induction, mineralized nodule formation was evaluated by 1% Alizarin Red S staining under a stereoscopic microscope.

### 2.4. Animals, Pain Model Establishment, and SHED Transplantation

Male Sprague-Dawley rats (8 weeks old, 200–250 g) were housed in a temperature-controlled room with free access to food and water. The trigeminal neuropathic pain model was established through CCI-ION using an intraoral approach [13]. The animals were anesthetized with pentobarbital sodium (50 mg/kg, intraperitoneally) during surgery. The head was fixed on a table in a supine position. A mucosal incision was made along the left maxillary gingivobuccal margin. The ION was loosely ligated with two chromic gut (4.0) ligatures. In sham rats, the unilateral infraorbital nerve was exposed without ligature. All experiments were approved by the Ethical Committee of Animal Research at China Medical University (G2019008).

Three days after nerve ligation, rats were injected once with low-dose (1 × 10^5^ cells, 0.2 mL, *n* = 6 rats, CCI-ION+low-dose injSHED group) or high-dose (1 × 10^6^ cells, 0.2 mL, *n* = 6 rats, CCI-ION+high-dose injSHED group) SHED at the lesion site. Rats in the CCI-ION+infSHED group received a single infusion of 1 × 10^5^ SHED (0.2 mL, *n* = 6) through the tail vein 3 days after nerve ligation. Control rats were injected with serum-free culture medium locally (CCI-ION+injMedium) or intravenously (CCI-ION+infMedium). All animals were killed with an overdose of sodium pentobarbital (70 mg/kg, intraperitoneally). Animals were perfused through the ascending aorta with saline, followed by 4% formaldehyde.

### 2.5. Behavioral Testing

The mechanical threshold was determined before and 3, 7, 14, 28, and 56 days after nerve ligation or SHED administration and was assessed by the same investigator under blind conditions. A series of calibrated von Frey hairs, with buckling weights between 2 g and 15 g, were applied to the skin above the ION with enough pressure to bend the filaments. An active withdrawal of the head from the von Frey hairs was defined as a positive response. Each von Frey filament was applied five times with intervals of a few seconds. The process was completed when the rats displayed 5 positive responses to the same filament. The response frequencies ([number of positive responses/number of total stimuli] × 100%) to a range of von Frey filament forces were calculated, and a stimulus-response frequency curve (*S*-*R* curve) was plotted. We used the EF50 value, defined as the von Frey filament force (*g*) that produces a 50% response frequency from the stimulus-response frequency curve, as a measure of the mechanical threshold. A decrease in EF50 indicates the presence of mechanical allodynia [14].

### 2.6. SHED Distribution

SHED were labeled with the PKH26 red fluorescent cell linker mini kit (Sigma-Aldrich, St. Louis, MO, USA), according to the manufacturer's instructions. Briefly, 1 mL Diluent C and 4 *μ*L PKH26 dye were added to cell pellets and incubated at room temperature for 5 min. The reaction was stopped with 5 mL of serum. Stained cells were centrifuged at 1200 rpm for 5 min and injected (1 × 10^6^ cells, 0.2 mL, *n* = 6 rats) at the lesion site three days after left ION ligation. The left TG, trigeminal spinal subnucleus caudalis and C1-C2 spinal cervical dorsal horn (VC/C2), rostral ventromedial medulla (RVM), heart, lung, liver, kidney, spleen, and intestine were collected 24 and 72 h after SHED transplantation. The tissues were fixed in 4% formaldehyde for 2 days, then dehydrated with 15% and 30% glucose in water, and cut into 10 *μ*m slices. Nuclei were stained with DAPI (1 *μ*L/mL) for 15 min at room temperature. PKH26 (red) and DAPI (blue) fluorescent signals were analyzed with an LSM 510 confocal laser scanning microscope (Carl Zeiss, Germany).

### 2.7. Hematoxylin and Eosin (HE) Staining

HE staining was employed to evaluate the effect of SHED on inflammatory cell infiltration at the injured nerve. Animals were killed 7 days after SHED transplantation. Paraffin-embedded trigeminal nerves of sham rats and CCI-ION rats with or without SHED transplantation were cut to a thickness of 10 micrometers, dehydrated twice with 100% ethanol for 10 min, and rinsed with water. Then, the sections were stained with Harris' hematoxylin solution (Baso, Zhuhai, China) for 3 min and rinsed with water. Ten percent acetic acid and 85% ethanol were used for tissue differentiation. Then, the sections were stained with eosin Y ethanol solution (Baso) for 2 min. The sections were dehydrated with two changes of 95% alcohol and two changes of 100% alcohol. After extracting the alcohol with two changes of xylene, the sections were covered with coverslips and observed and imaged under a light microscope (Olympus SZX12; Olympus, Tokyo, Japan).

### 2.8. Quantitative Reverse Transcription (RT-q) PCR

Animals were killed 7 days and 56 days after SHED transplantation. The mRNA levels of *TNF-α* and *IL-1β* in the trigeminal nerve were quantified by RT-qPCR using the Stratagene/SYBR green Supermix system. We used the PrimeScript RT reagent kit with gDNA Eraser to prevent contamination of genomic DNA. Total RNA was extracted from injured nerves using the TRIzol reagent (Invitrogen, Carlsbad, CA, USA) and was purified using a RNeasy kit (Qiagen, MD, USA). cDNA was generated from 1 *μ*g of RNA using SuperScript II (Invitrogen, Carlsbad, CA, USA) and 2.5 ng of random primer per reaction. The PCR program was as follows: 5 min at 94°C and 3 min at 95°C, followed by 30 cycles of 30 s at 95°C, 45 s at 58°C, and 20 s at 68°C. *β*-Actin was used as a housekeeping gene for normalization. The following primers were used: *TNF-α*, forward 5′-CCC CGA CTA TGT GCT CCT CAC-3′ and reverse 5′-GGG CTC TTG ATG GCG GA-3′; *IL-1β*, forward 5′-GGA AGG CAG TGT CAC TCA TTG TG-3′ and reverse 5′-GGT CCT CAT CCT GGA AGC TCC-3′; and *β-actin* (housekeeping gene for normalization), forward 5′-GGT CCA CAC CCG CCA CCA G-3′ and reverse 5′-CAG GTC CAG ACG CAG GAT GG-3′. All PCRs were run in triplicate. Relative mRNA levels were calculated using the comparative CT method (ΔΔCT method).

### 2.9. Immunohistochemical Staining

Animals were killed 7 days after SHED transplantation. After perfusion, the left TG and trigeminal nerves were collected and postfixed in 4% paraformaldehyde overnight. The tissue was sectioned coronally at 10 *μ*m. Conventional immunohistochemistry procedures were performed, using a primary antibody against TRPV1 (1 : 1000, Neuromics, Edina, MN, USA), followed by Cy-3-conjugated goat anti-rabbit antiserum (1 : 250, Jackson ImmunoResearch, West Grove, PA, USA). TRPV1-positive cells were counted from six representative sections per ganglion from 6 TGs. Only labeled neurons with a clear nucleus were evaluated. The TRPV1 staining intensity in the trigeminal nerve was analyzed using the ImageJ software (National Institutes of Health, MA, USA).

### 2.10. Western Blot

Animals were killed 7 days after SHED transplantation. Total proteins were extracted from the left TG and trigeminal nerves of sham rats, CCI-ION rats without SHED transplantation, and CCI-ION rats with SHED transplantation. Proteins were extracted with a tissue protein extraction reagent (Weiao Biotech, Shanghai, China), and 50 *μ*g of each sample was run on a polyacrylamide gel, transferred to a nitrocellulose membrane, blocked (Tris Buffered Saline with Tween (TBST) containing 5% fat-free dry milk) for 2 h at room temperature, and incubated overnight at 4°C with antibodies against TRPV1 (1 : 400; Santa Cruz). The membrane was washed and incubated with a horseradish peroxidase-conjugated secondary antibody (Santa Cruz Biotechnology) at 37°C for 2 h. The immunoreactivity was detected using ECL (Western Lightning, USA). Protein level for TRPV1 was normalized to that of *β*-actin in the same sample.

### 2.11. Statistical Analysis

All data are expressed as the mean ± standard error of the mean (SEM). Mechanical threshold data were analyzed using two-way repeated measures ANOVA followed by Bonferroni's post hoc test. One-way ANOVA was used to compare data in the three groups. The significance level was determined at *p* < 0.05.

## 3. Results

### 3.1. Characterization of Isolated SHED for Use in Transplantation Studies

**Figure 1:**
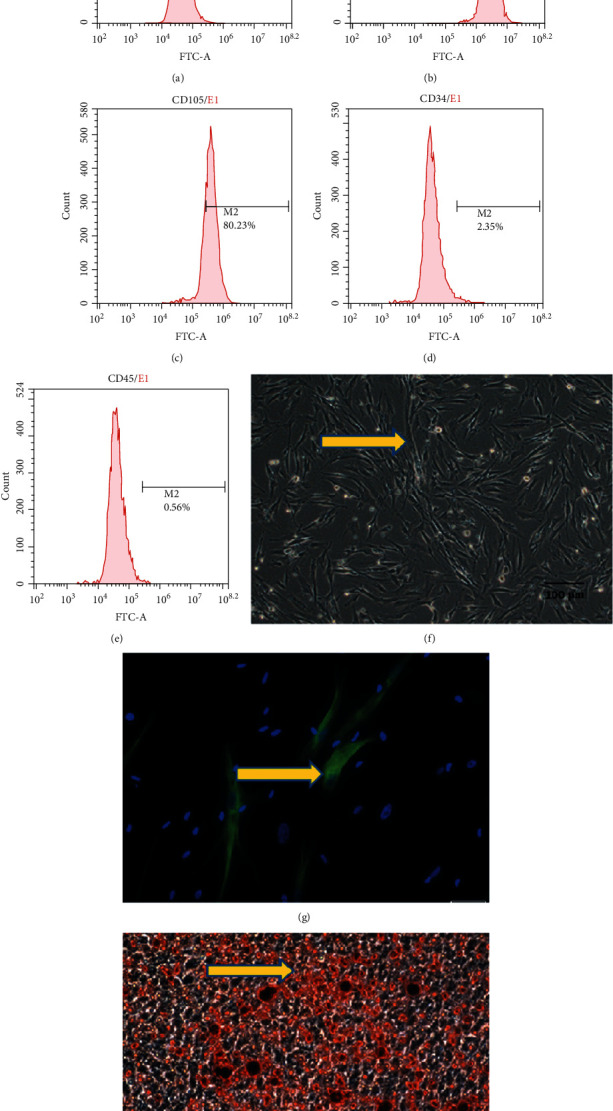
Characterization of stem cells isolated from human exfoliated deciduous teeth (SHED). Results of flow cytometric analyses of SHED are shown. (a) Isotype-matched IgG1 antibody was used as a control. Percentages of cell markers CD73 (b), CD105 (c), CD34 (d), and CD45 (e) expressed in SHED were analyzed by flow cytometry. (f) Morphologically, the cultured SHED were spindle-shaped. (g) Expression of the neural marker *β*III-tubulin in SHED after 4 weeks of neural inductive culture. (h) SHED formed Alizarin Red S-positive mineralized nodules after 4 weeks of induction of osteogenic differentiation. Scale bar = 20 *μ*m (g), scale bar = 100 *μ*m (f, h).

Flow cytometric analysis showed that 99.27% and 80.23% of the cultured SHED were positive for CD73 (Figure 1(a)) and CD105 (Figure 1(c)), respectively. Only 2.35% and 0.56% of the cells were positive for CD34 (Figure 1(d)) and CD45 (Figure 1(e)), respectively. These results indicated that SHED expressed a set of mesenchymal stem cell markers, but not hematopoietic stem cell markers. The cultured SHED were mostly spindle-shaped (Figure 1(f)).

After 4 weeks of neural inductive culture, the SHED expressed the neural marker *β*III-tubulin, indicating their neural differentiation potential (Figure 1(g)). Alizarin Red S-positive nodules formed in the SHED culture after 4 weeks of induction of osteogenic differentiation (Figure 1(h)), indicating the potential of SHED to differentiate into mineralized tissue *in vitro*.

### 3.2. Local Injection or Infusion of SHED Reverses Pain Hypersensitivity after CCI-ION

**Figure 2:**
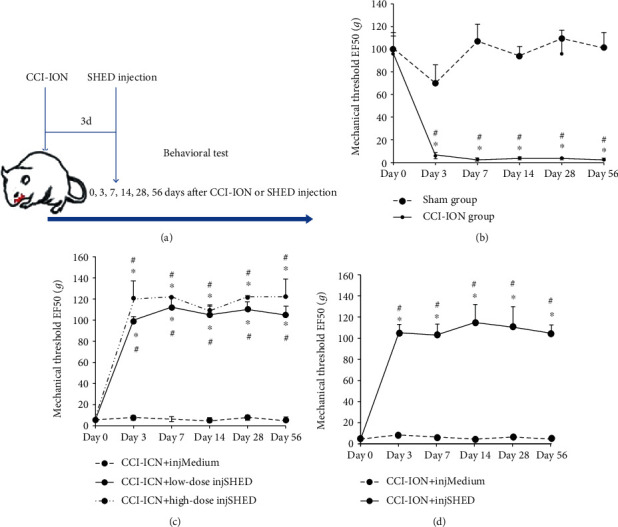
Effect of SHED transplantation on neuropathic pain. (a) Scheme showing the timing of SHED transplantation and behavioral testing. (b) The mechanical threshold was significantly decreased in CCI-ION rats. (c) Changes in the mechanical threshold after direct injection of low-dose (1 × 10^5^ cells, 0.2 mL, CCI-ION+low-dose injSHED group) or high-dose (1 × 10^6^ cells, 0.2 mL, CCI-ION+high-dose injSHED group) SHED into the site of injury were measured for 56 days in CCI-ION rats. Local injection of medium (CCI-ION+injMedium group) was used as a control. (d) Changes in the mechanical threshold after infusion of SHED (1 × 10^5^ cells, 0.2 mL, CCI-ION+infSHED group) through the tail vein were measured for 56 days in CCI-ION rats. Infusion of medium (CCI-ION+infMedium group) was used as a control. ^∗^*p* < 0.05 vs. sham (b), ^∗^*p* < 0.05 vs. medium (c, d), and ^#^*p* < 0.05 vs. day 0. All data are expressed as the mean ± SEM (*n* = 6 rats in each group).

The trigeminal nerve is the major sensory nerve innervating the facial skin. The three large branches from the TG are the ophthalmic nerve (V1), maxillary nerve (V2), and mandibular nerve (V3). We induced neuropathic pain through CCI of the infraorbital nerve, which is the major branch of V2. Two-way repeated measures ANOVA showed significant main effects for treatment (*p* = 0.009) and time (*p* < 0.001) and significant interaction (*p* < 0.001). In the CCI-ION group, the decrease in EF50 was significant (*p* < 0.001) at the first test on day 3 postligation. The mechanical allodynia lasted more than 8 weeks in this model, as indicated by the significant and persistent reduction in mechanical threshold 3, 7, 14, 28, and 56 days after nerve ligation (*p* < 0.001, *p* = 0.006, *p* = 0.002, *p* = 0.008, and *p* = 0.003, respectively) (Figure 2(a)).

In pilot experiments, we found that SHED at passages 3–5 effectively produced long-term pain relief, whereas SHED at high passages (>20) were not effective (data not shown).

First, we tested the effects of direct injection of SHED into the injured trigeminal nerve on behavioral signs in CCI-ION rats. As shown in Figure 2(b), both low-dose (*p* < 0.001 at all time points) and high-dose SHED (*p* < 0.001 at all time points) reversed the mechanical hypersensitivity and allodynia 3, 7, 14, 28, and 56 days after SHED local injection when compared with CCI-ION+injMedium. Culture medium had no significant effect on the injury-induced mechanical threshold.

Next, we tested whether a single infusion of SHED was effective. In rats that had received an infusion of SHED, the EF50 on day 3 after infusion was significantly increased when compared with that in the medium control (*p* < 0.001) and the baseline level (*p* < 0.001). The analgesic effects persisted throughout the observation period of 8 weeks, as indicated by the significant increase in the mechanical threshold 3, 7, 14, 28, and 56 days after SHED infusion as compared with CCI-ION+infMedium (*p* < 0.001 at all time points) (Figure 2(c)).

### 3.3. Localization of Locally Transplanted SHED in the Early Phase

**Figure 3:**
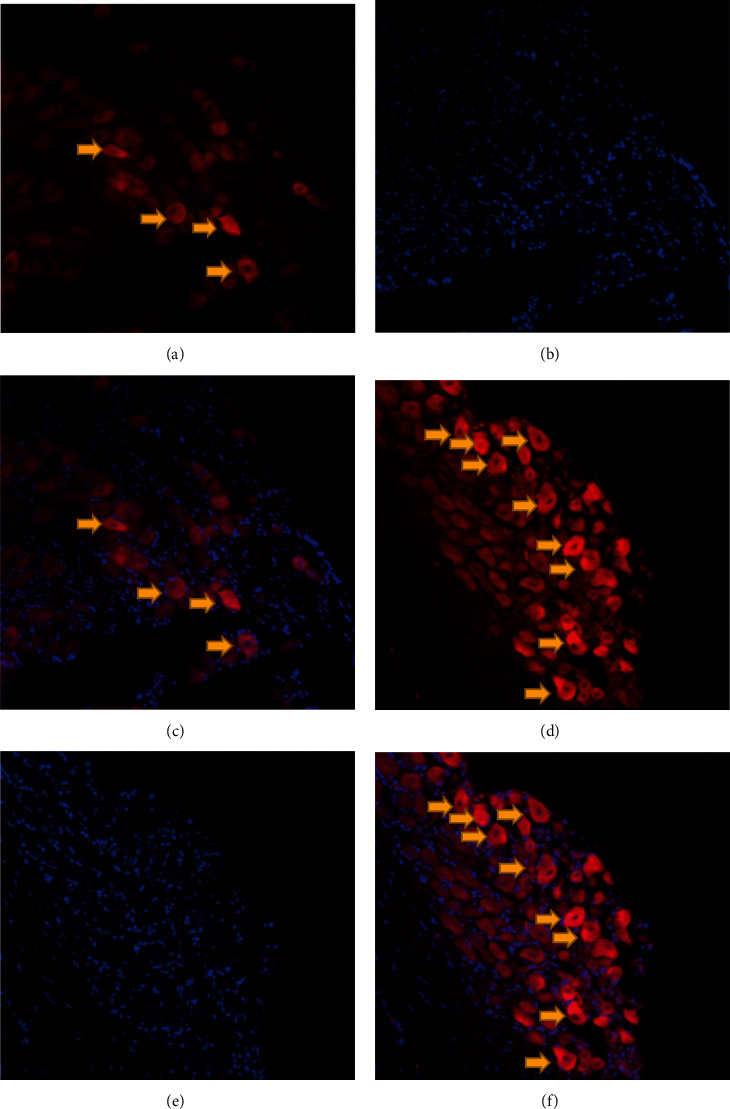
Distribution of PKH26-labeled SHED in the ipsilateral TG: (a) PKH26-positive cells (red) 24 h after local injection; (b) DAPI-stained cells (blue) 24 h after local injection; (c) the merged image of (a) and (b); (d) PKH26-positive cells (red) 72 h after local injection; (e) DAPI-stained cells (blue) 72 h after local injection; (f) the merged image of (d) and (e).

SHED stained with both PKH26 and DAPI were distributed to the ipsilateral TGs 24 h (Figures 3(a)–3(c)) and 72 h (Figures 3(d)–3(f)) after local injection. PKH26-positive cells were not found in the trigeminal spinal subnucleus caudalis and C1-C2 spinal cervical dorsal horn (VC/C2), rostral ventromedial medulla (RVM), heart, lung, liver, kidney, spleen, or intestine.

### 3.4. Local SHED Transplantation Inhibits Inflammatory Cell Infiltration in the Injured Nerve

**Figure 4:**
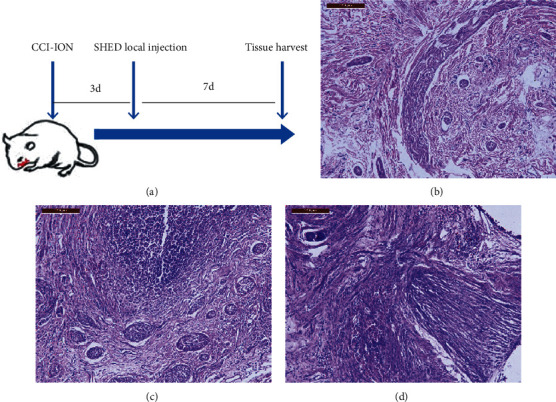
Local injection of SHED inhibits inflammatory cell infiltration in the injured nerve. (a) Scheme showing the timing of SHED transplantation and tissue collection. Hematoxylin-eosin staining revealed that the number of leukocytes recruited in the trigeminal nerve was significantly increased in the CCI-ION group (c) as compared with the sham group (b). Inflammatory cell infiltration in the trigeminal nerve was decreased after local injection of SHED (1 × 10^5^ cells, 0.2 mL) (d). Scale bar = 200 *μ*m.

Injured trigeminal nerves were examined histologically by HE staining 7 days after injection of SHED into the lesion site. As shown in Figure 4, the number of leukocytes recruited in the trigeminal nerve was significantly increased in the CCI-ION group (Figure 4(c)) as compared with the sham group (*p* < 0.001) (Figure 4(b)). However, inflammatory cell infiltration in the trigeminal nerve was decreased in the CCI-ION+low-dose injSHED group when compared with the CCI-ION+injMedium group (*p* < 0.001) (Figure 4(d)).

### 3.5. The Effect of Local SHED Transplantation on the Levels of TNF-*α* and IL-1*β* in the Trigeminal Nerve

**Figure 5:**
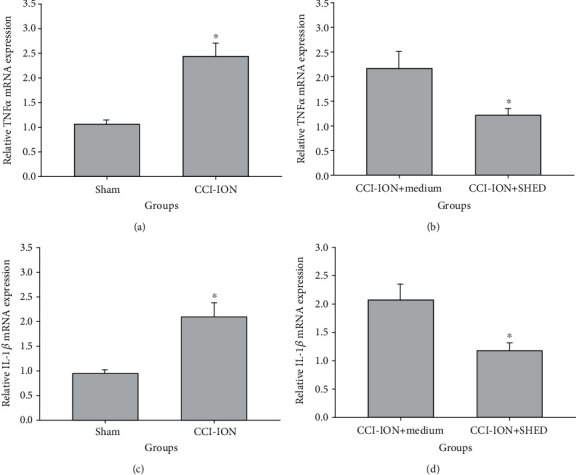
Local SHED transplantation reduces *TNF-α* and *IL-1β* mRNA levels in the trigeminal nerve 7 days after transplantation. mRNA levels of *TNF-α* (a) and *IL-1β* (c) in the injured trigeminal nerve measured 7 days after CCI-ION. mRNA levels of *TNF-α* (b) and *IL-1β* (d) in the injured trigeminal nerve measured 7 days after local SHED transplantation (1 × 10^5^ cells, 0.2 mL). ^∗^*p* < 0.05 vs. sham (a, c), ^∗^*p* < 0.05 vs. medium (b, d). All data are expressed as the mean ± SEM (*n* = 6 rats in each group).

To evaluate the effect of SHED transplantation on the expression of proinflammatory cytokines, we examined the *TNF-α* and *IL-1β* mRNA levels in the injured trigeminal nerves by RT-qPCR 7 and 56 days after local transplantation. The CCI-ION group showed significantly higher mRNA levels of *TNF-α* (*p* = 0.002, Figure 5(a)) and *IL-1β* (*p* = 0.011, Figure 5(c)) than the sham group 7 days after local transplantation. However, local SHED transplantation reduced the mRNA levels of *TNF-α* (*p* = 0.049, Figure 5(b)) and *IL-1β* (*p* = 0.025, Figure 5(d)) in injured nerves as compared with the medium control 7 days after local transplantation. These results suggest that SHED significantly inhibited the CCI-ION-induced upregulation of proinflammatory cytokine in injured nerves in the neuropathic rats in the early phase.

**Figure 6:**
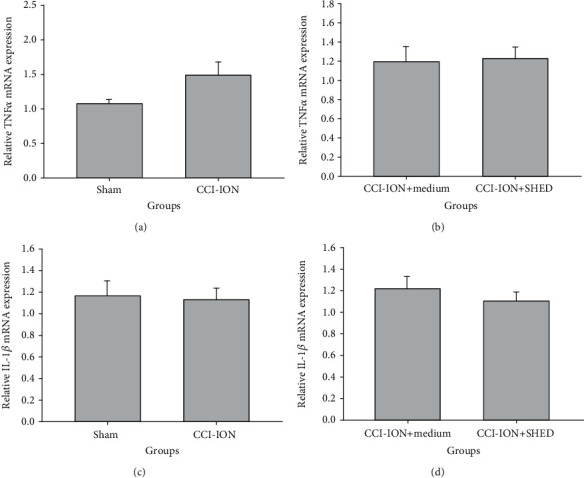
Local SHED transplantation reduces *TNF-α* and *IL-1β* mRNA levels in the trigeminal nerve 56 days after transplantation. mRNA levels of *TNF-α* (a) and *IL-1β* (c) in the injured trigeminal nerve measured 56 days after CCI-ION. mRNA levels of *TNF-α* (b) and *IL-1β* (d) in the injured trigeminal nerve measured 56 days after local SHED transplantation (1 × 10^5^ cells, 0.2 mL). ^∗^*p* < 0.05 vs. sham (a, c), ^∗^*p* < 0.05 vs. medium (b, d). All data are expressed as the mean ± SEM (*n* = 6 rats in each group).

There were no significant differences in the mRNA levels of *TNF-α* (Figure 6(a)) and *IL-1β* (Figure 6(c)) in the injured trigeminal nerves between the sham and the CCI-ION groups 56 days after nerve ligation (*p* > 0.05). Local SHED transplantation did not change the *TNF-α* (Figure 6(b)) and *IL-1β* mRNA levels (Figure 6(d)) in the injured trigeminal nerves 56 days after SHED injection (*p* > 0.05).

### 3.6. Local SHED Transplantation Suppresses Upregulation of TRPV1 Expression in CCI-ION Rats

**Figure 7:**
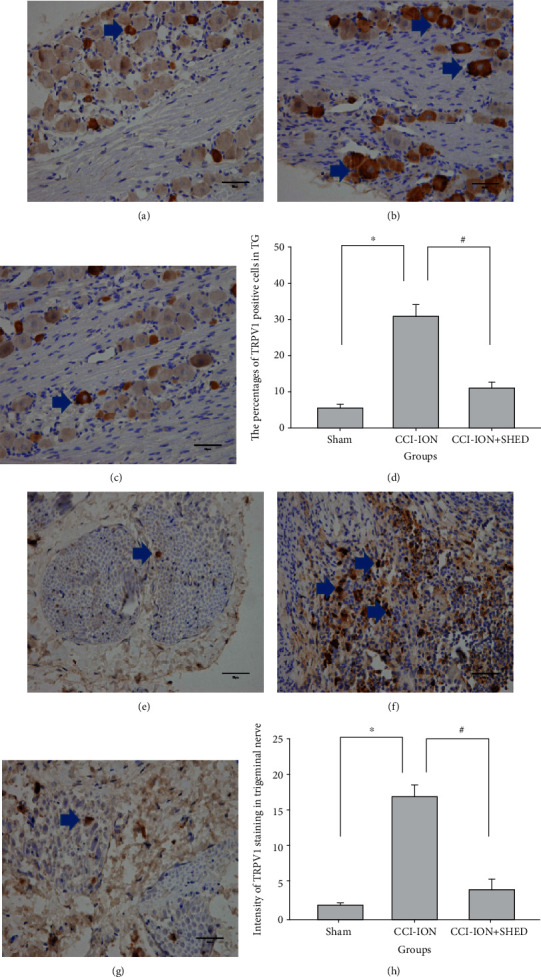
Local SHED transplantation suppresses upregulation of TRPV1-positive cells in CCI-ION rats. TRPV1-positive cells in the TG (a) and trigeminal nerve (e) of sham rats. TRPV1-positive cells in the TG (b) and trigeminal nerve (f) of CCI-ION rats without SHED injection. TRPV1-positive cells in the TG (c) and trigeminal nerve (g) of CCI-ION rats with local SHED administration (1 × 10^5^ cells, 0.2 mL). (d) Quantification of TRPV1-positive cells in the TG. (h) Quantification of TRPV1 staining in the trigeminal nerve. Scale bar = 50 *μ*m. ^∗^*p* < 0.05 vs. sham, ^#^*p* < 0.05 vs. CCI-ION rats without SHED transplantation. All data are expressed as the mean ± SEM (*n* = 6 rats in each group).

**Figure 8:**
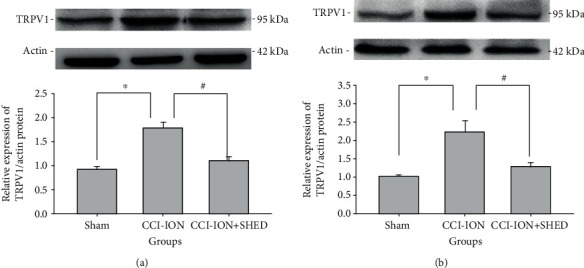
Local SHED transplantation suppresses upregulation of TRPV1 protein levels in CCI-ION rats. TRPV1 protein expression in the TG (a) and trigeminal nerve (b) of sham rats. All data are expressed as the mean ± SEM (*n* = 6 rats in each group).

Trigeminal nerves and TGs were extracted 7 days following local SHED injection. TRPV1 expression was examined by immunohistochemical staining (Figure 7) and western blot (Figure 8). TRPV1-positive cells were substantially increased in the TG (*p* < 0.001) and the trigeminal nerve (*p* < 0.001) in the CCI-ION group as compared with the sham rats (Figure 7). CCI-ION-induced upregulation of TRPV1 expression in the TG (*p* < 0.001) and the primary afferent nerve (*p* < 0.001) was significantly suppressed by local SHED administration (Figure 7).

TRPV1 protein levels in the TG (*p* < 0.001) and trigeminal nerve (*p* = 0.003) after nerve ligation were upregulated compared with those in sham rats (Figure 8). Local SHED transplantation inhibited CCI-ION-induced upregulation of TRPV1 expression in the TG (*p* < 0.001) and the primary afferent nerve (*p* = 0.011) (Figure 8).

## 4. Discussion

The present study demonstrated that systemic or local injection of SHED attenuated the sensitivity to mechanical stimuli in rats after trigeminal nerve injury, and this effect lasted throughout the observation period of 8 weeks. PKH26-labeled SHED were distributed to the ipsilateral TG 24 and 72 h after local injection. SHED transplantation at the lesion site reduced inflammatory cell infiltration and proinflammatory cytokine levels in the injured nerve in the early phase. Moreover, local SHED transplantation inhibited CCI-ION-induced upregulation of TRPV1 expression in the trigeminal nerve and ganglion.

The use of dental-derived mesenchymal stem cells in tissue repair or reconstruction is considered an intriguing milestone for translational regenerative medicine. Dental-derived mesenchymal stem cells have a strong ability to differentiate into neural, osteogenic, and adipogenic lineages [15–17]. For example, SHED transplantation provided nerve protection by increasing nerve growth factor protein levels and the capillary density in diabetic rats [18]. Recent studies have revealed that bone marrow stromal cells (BMSCs) reduce the progress of neuropathic pain. Systemic and local administration of BMSCs attenuated mechanical and thermal hyperalgesia in various neuropathic pain models. Intravenous injection of BMSCs reverted nociceptive hypersensitivity in CCI-ION models [19, 20] and sciatic nerve CCI models [21]. BMSCs have been shown to produce pain relief upon intrathecal [22], intrabrain [23], and intraspinal [24] injection. When BMSCs were injected directly into the lesion site, the mechanical and thermal thresholds were increased in CCI-ION rats, CCI models of the sciatic nerve, and spinal cord injury models [20, 25, 26].

SHED reportedly show a higher proliferation rate and basic fibroblast growth factor gene expression than adult BMSCs and dental pulp stem cells [2, 27, 28]. SHED can be easily obtained, without the need for invasive procedures, and thus represent a large source of stem cells for potential clinical application, without ethical issues. More importantly, as neural crest cell-associated postnatal stem cells, SHED express various neural cell markers [2], release key neurotrophic factors, and suppress the apoptosis of astrocytes and neurons [7]. Importantly, SHED transplantation relieved diabetic peripheral neuropathy and increased the capillary density in diabetic rats [18]. Therefore, SHED may have advantages over BMSCs in pain control and potentially become an alternative and efficacious treatment for neuropathic pain.

It remains unclear how stem cells inhibit pain. It has been suggested that stem cells may reduce pain through a cell-to-cell contact activation mechanism or the secretion of various molecules such as cytokines, regulating the local environment in many pathological conditions [21, 29, 30]. Growing evidence suggests that the majority of systemic BMSCs are rapidly trapped in the lungs and their long-lasting antihyperalgesic effect is substantially longer than their survival time in the host [31, 32]. Thus, the topical mechanism of BMSCs in chronic pain management is the secretion of various molecules to inhibit neuroinflammation and modulate cytokine levels. For example, in sciatic nerve CCI model mice [22, 33], intrathecal BMSCs exerted a persistent analgesic effect through the release of the anti-inflammatory growth factor transforming growth factor beta, which inhibited CCI-induced spinal neuroinflammation, downregulated TNF-*α* expression, and modulated excitatory synaptic transmission in spinal cord and dorsal root ganglion neurons [22, 33]. Nuclear factor kappa B (NF-*κ*B) signaling plays a key role in BMSC-induced antihyperalgesia in rat models of persistent pain and spinal cord injury [34, 35]. In spinal cord injury rats, BMSCs downregulated phosphorylated NF-*κ*B P65 subunit and decreased other proinflammatory factors, such as TNF-*α* and IL-1*β* [35]. Human BMSCs attenuated mechanical allodynia and thermal hyperalgesia by reducing the levels of the proinflammatory cytokines IL-1*β* and IL-17 and releasing the anti-inflammatory cytokine IL-10 in a mouse spared nerve injury model of neuropathic pain [21, 23].

In support of the mechanism of BMSCs in pain control, our study provided evidence that local SHED transplantation attenuates neuropathic pain by reducing inflammatory cell infiltration and proinflammatory cytokine levels in the injured nerve in the early phase. There is sufficient evidence to show that cytokines play an important role in the inflammatory response to neuropathic pain. A reduction in proinflammatory cytokine levels elevated by nerve injury contributes to pain relief [36, 37].

It is relevant to mention that SHED possess immunomodulatory properties and regulate cytokine levels in other conditions. SHED inhibit lymphocyte stimulation, accompanied by a decrease in proinflammatory factors (TNF-*α* and IL-2) and an increase in anti-inflammatory factors (IL-6 and IL-10) [38]. Additionally, SHED modulate the immune system and relieve immune disorders, such as SLE [8]. SHED transplantation reduced proinflammatory cytokine IL-17 levels and upregulated the ratio of regulatory T cells to helper T 17 cells *in vitro* and reversed SLE-associated disorders and improved SLE phenotypes in mice [8]. Further studying the effects of SHED on immune responses to nerve injury will be a promising direction toward understanding the mechanisms underlying the analgesic effects of SHED.

Another important finding of this study is that SHED transplantation inhibited CCI-ION-induced upregulation of TRPV1 expression in the primary afferent nerve and TG, which may suggest that TRPV1 is involved in the strong analgesic effect of SHED. TRPV1 is a key molecular component of pain detection and modulation. In neurons, TRPV1 activation increases the calcium influx and contributes to a higher perception of pain [9–11]. In this work, TRPV1 expression in the trigeminal nerves at the injury site and ipsilateral ganglia was increased after nerve injury in rats, which suggests that peripheral TRPV1 expression mediates trigeminal neuralgia. In line herewith, previous studies reported that TRPV1 expression was upregulated in nerve fibers and ganglia following spinal and cranial nerve injury. In sciatic nerve ligation rats, TRPV1 mRNA and protein levels in the dorsal root ganglion and spinal dorsal horn were increased up to 1–4 weeks after injury [39, 40]. TRPV1 expression was upregulated in the injured nerves and TG neurons 3 weeks after lingual nerve transaction in adult ferrets [41]. In mice, following trigeminal injury, the somata of numerous large neurons in the injured V2 can be activated by the TRPV1 ligand, capsaicin [9]. TRPV1 hyperactivity has been observed in central fibers and terminals of TG neurons innervating all dorsal horn lamina in mice after CCI-ION. Our data showed that SHED transplantation reversed neuropathic pain through downregulation of peripheral TRPV1 expression and regulation of neuronal plasticity. However, the molecular mechanisms by which SHED regulate TRPV1 expression are unknown and require further investigation. One limitation of this study is that we did not study the relationship between cytokines and TRPV1 expression in SHED-mediated analgesia. In future, we will focus on the detailed mechanisms of SHED in relieving neuropathic pain.

## 5. Conclusions

This study revealed a novel role of SHED in reversing hypersensitivity and allodynia in rats after trigeminal nerve injury. Paracrine function and downregulation of TRPV1 expression in the peripheral nervous system may be the mechanisms underlying SHED-based analgesia. These results provide preclinical evidence supporting the use of dental stem cells in the treatment of trigeminal neuralgia and potentially other chronic pain conditions.

## Data Availability

The data used to support the findings of this study are available from the corresponding author upon request.
